# Stakeholder Attitudes on AI Integration in Ophthalmology

**DOI:** 10.1055/a-2543-4330

**Published:** 2025-04-16

**Authors:** Amr Saad, Ferhat Turgut, Matthias Becker, Delia DeBuc, Gabor Mark Somfai

**Affiliations:** 1 Department of Ophthalmology, Stadtspital Zurich Triemli, Zurich, Switzerland; 2 Spross Research Institute, Zurich, Switzerland; 3 Ophthalmology, Gutblick, Pfäffikon, Switzerland; 4 Department of Ophthalmology, University of Heidelberg, Germany; 5 Bascom Palmer Eye Institute, University of Miami School of Medicine, Miami, FL, USA; 6 Department of Ophthalmology, Semmelweis University, Budapest, Hungary

**Keywords:** artificial intelligence, ophthalmology, implementation, künstliche Intelligenz, Augenheilkunde, Implementierung

## Abstract

Artificial intelligence (AI) is gaining widespread traction in ophthalmology, with multiple screening and diagnostic tools already being approved by U. S. and EU authorities. However, the adoption of these tools among medical professionals and their acceptance among patients is still questionable. This narrative review analyses the current literature on stakeholder perspectives on the integration of AI in ophthalmology, with a focus on comparing views across different global healthcare contexts. A PubMed search was conducted for original research articles published between January 1, 2015 and August 31, 2024. The analysis revealed different levels of acceptance for different AI applications among different stakeholder groups. Ophthalmologists and optometrists generally showed positive attitudes toward AI as an adjunct tool, while patients expressed mixed views, appreciating potential benefits while expressing concerns about a lack of transparency in the integration of AI into healthcare. This review reveals a complex landscape of stakeholder perspectives on AI in ophthalmology, highlighting the need for tailored approaches to AI implementation that address specific concerns and consider different healthcare contexts. The findings underscore the importance of collaborative efforts to develop context-specific, effective AI solutions in ophthalmology.

## Zusammenfassung

Der Einsatz Künstlicher Intelligenz (KI) in der Augenheilkunde erfährt eine zunehmende Beliebtheit. Mehrere Screening- und Diagnoseinstrumente wurden bereits von den US-amerikanischen und europäischen Behörden zugelassen. Die Akzeptanz dieser Anwendungen durch das medizinische Fachpersonal und die Patienten ist jedoch noch unklar. In dieser Übersichtsarbeit wird die aktuelle Literatur zu den Perspektiven der Stakeholder bez. der Integration von KI in die Augenheilkunde analysiert. Es wurde eine PubMed-Suche nach Original-Forschungsartikeln durchgeführt, die zwischen dem 1. Januar 2015 und dem 31. August 2024 veröffentlicht wurden. Die Analyse ergab eine unterschiedliche Akzeptanz verschiedener KI-Anwendungen bei den verschiedenen Interessengruppen. Augenärzte und Optometristen zeigten eine generell positive Einstellung gegenüber KI als ergänzendes Instrument, während Patienten eine gemischte Meinung äußerten, indem sie einerseits die potenziellen Vorteile schätzten, andererseits aber auch Bedenken hinsichtlich der mangelnden Transparenz bei der Integration von KI in die Gesundheitsversorgung äußerten. Dieser Überblick zeigt eine komplexe Landschaft von Stakeholder-Perspektiven zu KI in der Augenheilkunde und unterstreicht die Notwendigkeit maßgeschneiderter Ansätze zur Implementierung von KI, die auf spezifische Bedenken eingehen und unterschiedliche Kontexte im Gesundheitswesen berücksichtigen. Die Ergebnisse unterstreichen die Bedeutung gemeinsamer Anstrengungen zur Entwicklung kontextspezifischer, effektiver KI-Lösungen in der Augenheilkunde.

## Introduction

The integration of artificial intelligence (AI) in healthcare has been proclaimed to be a revolutionary force with the potential to transform patient care, enhance diagnostic precision, and optimize clinical workflows 1 . With its substantial reliance on imaging and pattern recognition, ophthalmology has emerged as a pioneering field in the adoption and development of AI applications 2 . From automated screening for diabetic retinopathy to AI-assisted interpretation of optical coherence tomography (OCT) scans, the field has witnessed a surge in AI-driven innovations over the past few years 3 .

As AI technologies evolve and become more present in various aspects of ophthalmic practice, it becomes increasingly important to understand the perspectives of key stakeholders, including ophthalmologists, optometrists, patients, and healthcare organizations. These perspectives not only influence the future of AI adoption but also inform the development of ethical frameworks, regulatory policies, and implementation strategies in the future 4 .

Recent survey-based studies have provided insights into the complex landscape of AI perception in ophthalmology. The global landscape of AI adoption in ophthalmology is far from uniform, highlighting the need for a nuanced, context-specific approach to AI implementation that considers the unique challenges and opportunities within diverse healthcare systems 5 . This review aims to give a thematic analysis and synthesize the existing literature on AI perception in ophthalmology, with a particular focus on comparing the views of different stakeholders.

## Methods

A comprehensive search of electronic databases, including PubMed and MEDLINE, was conducted to identify relevant literature. The search strategy employed a combination of key words related to AI, ophthalmology, and stakeholder perspectives. The search string included the following terms, which were combined using Boolean operators: (“artificial intelligence” OR “machine learning” OR “deep learning”) AND (“ophthalmology” OR “eye care”) AND (“perspective” OR “survey” OR “questionnaire”). To ensure the inclusion of the most recent developments in the field, the search was limited to articles published in English between January 1, 2015 and August 31, 2024. Only peer-reviewed articles reporting survey-based studies on perceptions of AI in ophthalmology and studies involving ophthalmologists, optometrists, patients, or healthcare organizations were included in the review. A thematic analysis approach was employed to synthesize the data collected from the selected research articles, along with a categorization of stakeholders.

## Brief Overview of the Current Landscape of Artificial Intelligence Applications in Ophthalmology

The integration of AI into the domain of ophthalmology has shown a notable and rapid development in recent years, significantly enhancing diagnostic capabilities, treatment planning, and patient management 2 .

Convolutional neural networks (CNNs) have demonstrated remarkable performance in the detection and classification of diabetic retinopathy (DR), with high accuracy of over 95% and 0.97 area under the curve (AUC) 6 , 7 . A number of deep learning algorithms (DLA) have already received approval from the Food and Drug Administration (FDA), including IDx-DR, Eyenuk, and the AEye system, which also achieve sensitivity and specificity metrics of around 90% 3 , 8 . In addition, DeepMindʼs collaboration with Moorfields Eye Hospital has led to the development of an AI system capable of recommending treatment decisions for over 50 retinal diseases with a very high accuracy and an AUC of 99% for most pathologies 9 . In the field of glaucoma management, AI algorithms have demonstrated efficacy in analyzing OCT images and visual fields, demonstrating an AUC of 0.97 and outperforming most human experts 10 .

The application of AI in cataract surgery planning has gained significant traction, with systems like the Barrett Universal II formula incorporating AI algorithms to improve intraocular lens (IOL) power and others to refine effective lens position (ELP) calculations. These have demonstrated superior accuracy to traditional methods, particularly in eyes with extreme axial lengths 11 , 12 .

The advancement of AI has the potential to positively impact pediatric ophthalmology as well, particularly in the screening for retinopathy of prematurity (ROP). The i-ROP DL system, for instance, can reduce the burden on specialists in resource-limited settings 13 . Additionally, AI has shown promise in ocular oncology, notably in differentiating benign from malignant intraocular tumors. This includes the classification of choroidal nevus and melanoma based on multimodal imaging 14 .

## Stakeholder Perspectives on Artificial Intelligence in Ophthalmology

The emergence of potential AI applications in ophthalmology has evoked a range of responses from various stakeholders, with concerns, expectations, and insights varying across groups. In this context, the primary stakeholders can be broadly classified into two categories: (1) ophthalmologists and other eye care professionals, and (2) technology developers, patients, healthcare administrators, policymakers, and regulatory bodies. Understanding these perspectives is crucial for facilitating the effective adoption and implementation of AI technologies in clinical practice 4 .

The multinational Acceptance and Perception of Artificial Intelligence Usability in Eye Care (APPRAISE) study was conducted in 2022 in the USA. The study results offer valuable insights into the perspectives of ophthalmologists, a pivotal stakeholder group, regarding integrating AI in ophthalmology across a spectrum of eye conditions and clinical contexts 15 . These insights encompass three levels: individual practitioners, organizational, and system-level policies and screening services, which are collectively termed the macrosystem. The findings suggest a preference for AI to be positioned as a supplementary tool that enhances cliniciansʼ expertise rather than as a substitute for their decision-making or diagnostic roles. The surveyed ophthalmologists indicated that they perceived AI to be most relevant for detecting DR, followed by glaucoma and age-related macular degeneration. This is consistent with the current state of AI development and the availability of validated tools in ophthalmology, where significant progress has been made in the automated detection of these major eye diseases. Notably, the COVID-19 pandemic served to accelerate the inclination towards integrating AI in clinical practice. While the significant benefit of AI applications included improved access to eye care services, concerns about medical liability from AI errors and the associated lack of regulatory clarity demonstrated the existence of barriers to its adoption. Additional concerns include the prospect of a shift in control over healthcare from medical professionals to profit-oriented corporations 16 . Furthermore, according to the study results, numerous decision-making processes of the DLA are opaque to medical professionals and challenging to comprehend, which ultimately results in a deficit of trust among ophthalmologists 17 .

A survey from Australia and New Zealand confirmed that ophthalmologists recognize the influential role of AI in improving disease screening and streamlining repetitive tasks, compared to professionals in dermatology and radiology. Almost three-quarters of the 632 respondents (71%) believe that AI will bring improvement in their field, with an even higher proportion (86%) being convinced about its impactful changes within a decade. However, there remains a need for increased education and specialty training to support ophthalmologists in navigating the integration of this new technology into clinical practice 16 .

Pediatric ophthalmologists have a generally positive view of the role of AI in their specialty, while recognizing the need to address concerns about performance, clinical integration, and training to realize the full potential benefits of AI in ophthalmology 18 . In this online survey study, 80 members of the American Association for Pediatric Ophthalmology and Strabismus expressed concerns about the impact on the clinical decision-making process, and a quarter believe that AI could interfere with the patient–physician relationship. This is broadly consistent with findings from the aforementioned broader APPRAISE survey of ophthalmologists across different subspecialties 15 . The common opinion of the Australian- and U. S.-based surveys was that as AI becomes more integrated into clinical practice, ophthalmologists believe it will be critical for the next generation of ophthalmologists to be equipped with the knowledge and skills to handle these tools responsibly 16 , 18 .

Another study among the members of the American Academy of Optometry (AAOP, n = 400) provides insight into optometristsʼ perspectives on the use of AI in ophthalmology and shows that while a significant proportion of optometrists surveyed had concerns about the diagnostic accuracy of AI, the majority agreed that AI will improve their daily practice, similar to ophthalmologists 19 . From an educational perspective, the majority (80.3%) of optometrists felt that AI should be incorporated into optometry schools and curricula, suggesting a strong desire for more education and exposure to AI technologies 19 . Like ophthalmologists, optometristsʼ willingness to incorporate AI into clinical practice increased from 53.3% before the COVID-19 pandemic to 65.5% after the pandemic. An important factor behind this could be the drive through the CPT code for AI-based screenings in the U. S., billable by both optometrists and ophthalmologists at a price of US$55. They also identified promising use cases such as disease screening, disease progression monitoring, and triage but were less likely to believe that AI should be used to make diagnosis or treatment decisions, fearing over-reliance on technology and a negative impact on the patient–provider relationship. The results of the survey by Ho et al. indicate that Australian optometrists are generally open to incorporating AI into their practice but want to see robust validation through high-quality clinical trials and against a retinal specialist, and a clear value proposition for patients before widespread adoption 20 . Addressing cliniciansʼ concerns about loss of autonomy and the relationship between AI and humans will also be important for successful implementation. Interestingly, attitudes were not significantly influenced by most individual or workplace characteristics, including age, gender, and practice location.

While optometrists and ophthalmologists may have different perspectives on clinical practice in ophthalmology, concerns regarding AI implementation are similar. Scanzera et al. found in their study mentioned above, that 53% of optometrists surveyed from the AAOP had doubts about the diagnostic accuracy of AI, while Valikodath et al. reported that 45% of U. S. ophthalmologists expressed similar concerns 18 , 19 . Despite the generally high willingness of ophthalmologists to adopt AI tools, they still express concerns about the high costs associated with implementing AI technology in ophthalmology practices, potential displacement, and compromising their high-value professional roles in the future, as discussed by Constantin et al 21 . Scanzera et al. found that 25.1% of optometrists were concerned that AI could replace them. At the same time, Valikodath et al. reported that 15% of pediatric ophthalmologists expressed fears that AI could negatively impact the healthcare workforce 18 , 19 . The authors emphasized the importance of maintaining their roles and responsibilities in patient care rather than being diminished by AI.

A systematic review confirming the presented findings of the generally positive perceptions of eye care professionals, including ophthalmologists and optometrists, also highlighted the need for close collaboration between key stakeholders, including AI developers and clinicians, to ensure that the technology effectively benefits providers and patients 22 .

A study by Wood et al. revealed significant discrepancies in the levels of AI awareness and engagement among key stakeholders, including students, academic staff, and institutions at the Medical College of Georgia at Augusta University (USA) 23 . Most students and faculty members reported that they learned about AI through media exposure, suggesting a notable lack of teaching on AI within the formal educational or professional channels of ophthalmic training. In addition, clinical faculty members indicated a greater preference for training in using AI in educational contexts. In contrast, students expressed a greater interest in training in the use of AI in clinical settings. This divergence in priorities illustrates the different perspectives and needs of these two key stakeholder groups. The authors emphasize the need to establish multidisciplinary teams that include experts in the education of practical AI applications and their potential benefits in healthcare. This highlights the critical role of institutional support and resources in enabling AIʼs effective and sustainable integration into ophthalmology education. Therefore, several initiatives are currently underway that propose the development and integration of AI curricula into the education of medical students and ophthalmologists 24 , 25 . From the perspective of healthcare organizations, high costs and a lack of evidence, experience, and resources to develop their own AI systems may hinder the seamless adoption of emerging technologies in their practice 26 , 27 .

While there are no comprehensive surveys assessing AI adoption in ophthalmology from the patient perspective, surveys that have assessed glaucoma patientsʼ acceptance of telemedicine approaches indicate a general openness to digital solutions for monitoring and consultation in chronic disease settings, including willingness to self-monitor, which can be seen as a first step for digital use on the way to acceptance of using AI tools 28 . This positive evolution may have been influenced by the COVID-19 pandemic, which forced the adoption of digital technologies in different areas of life, as the authors hypothesized. On the other hand, out-of-the-box AI models need further development and adaptation to be used on the patient side, as Cappellani et al. could show in their investigation on how ChatGPT can be used to answer patientsʼ questions appropriately 29 .

A survey of patients at Yale University and Weill Cornell (USA) revealed a high level of acceptance regarding the potential of AI to enhance healthcare in general, with over 55% of respondents indicating that they believe AI will have a positive impact 30 . However, patients expressed a strong desire for transparency, with two-thirds deeming it “very important” to be informed when AI plays a significant role in their care. Additionally, patients displayed varied comfort levels with AI depending on the clinical application and had notable concerns about issues like data privacy, reduced clinician interaction, and increased costs. This aligns very well with the concerns raised by healthcare professionals, including ophthalmologists and optometrists.

A systematic review of the clinical use of AI, which included the perceptions of various stakeholders and was not limited to ophthalmology, revealed a substantial underrepresentation of patient perspectives in existing scientific surveys 17 . This underrepresentation allows only for limited insights. According to the authors, although a diverse range of stakeholders influences AI implementation, the scarcity of patient-centric qualitative studies highlights a crucial avenue for future research. They express the imperative need to address this gap to ensure that AI tools are developed and integrated in a manner that aligns with patient expectations, enhances patient satisfaction, and preserves the integrity of patient–clinician interactions.

Mathenge et al. found in the RAIDERS randomized trial that immediate feedback on AI-supported DR screenings significantly increased referral adherence and acceptance by 30% compared to delayed human grading in a low-resource setting, resulting in a more rapid treatment start in Rwanda 31 . These findings demonstrate, in addition to greater acceptance and integration of AI tools in routine eye care delivery, that the technology can have a positive impact on the minimization of barriers to accessing eye care.

Although there is a lack of surveys assessing policymakersʼ attitudes towards AI integration in healthcare, particularly in ophthalmology, the systematic review by Wolf et al. has revealed a lack of high-quality publications examining the economic impact of AI in healthcare. This scarcity of evidence makes it challenging for policymakers to make well-informed decisions regarding adopting AI technologies 32 .

Despite the fact that ophthalmology is one of the leading fields in AI application, Ruamviboonsuk et al. emphasize the significance of comprehending the financial implications of AI, including both immediate costs (research, development, and validation) and long-term expenses (maintenance and upgrades) 27 . Additionally, they underscore the importance of evaluating the cost-effectiveness and quality of life enhancements associated with AI applications. Furthermore, the authors indicate that the existing reimbursement structures may be inadequate to encourage the widespread adoption of this technology by primary care providers. This underscores the necessity of aligning the economic viability of AI solutions with the needs and constraints of healthcare providers. Additionally, the review suggests that the costs of AI in ophthalmology should be benchmarked against not only human experts but also other AI-based solutions to ensure fair and competitive pricing.

Importantly, to drive successful adoption of AI in ophthalmology, explainable AI (XAI) is essential. XAI makes AI decisions transparent, allowing both medical professionals and patients to understand how diagnostic outcomes are derived. This transparency is critical in gaining trust among ophthalmologists and optometrists, as they need to explain AI-based recommendations to patients. It also facilitates regulatory compliance, clinical validation, and ethical considerations, which are crucial for broader acceptance. With more interpretable AI, clinicians can assess the reliability of AI predictions and ensure that the tools align with clinical reasoning and judgment, leading to better integration into medical workflows.

Additionally, placing patients at the center of AI adoption is vital. Ensuring that AI tools are patient-centric involves addressing patientsʼ concerns, improving their understanding of AIʼs role in making a diagnosis, and ensuring that AI enhances, rather than replaces, the human touch in healthcare. Building trust requires aligning AI tools with patient needs, privacy expectations, and clinical outcomes that resonate with patientsʼ priorities. Engaging patients in the development, testing, and deployment of AI tools can help tailor solutions that are not only technologically robust but also culturally and contextually relevant, thus enhancing adoption and satisfaction. Also, AI tools should seamlessly integrate with electronic health records (EHRs) and other clinical management systems to prevent workflow disruptions. Smooth interoperability enhances adoption by making it easier for healthcare professionals to use AI alongside traditional tools.

These results highlight a pressing need to address the global disparities in developing and implementing AI technologies in healthcare. The goal of achieving equitable access to these transformative tools will necessitate deliberate actions to diversify training data, develop local capacity for health informatics and data science, and cultivate international collaborations. This approach will ensure that AI-powered solutions are tailored to distinctive needs and contexts of underserved regions. Nakayama et al. if highlighted that the current AI datasets in ophthalmology are heavily skewed towards a few developed countries 5 . This has resulted in the creation of biased AI models that may perform poorly when deployed in low-income and low to middle income countries, where the burden of preventable blindness is the highest. This issue has also been emphasized by others 5 , 33 .

While the literature reviewed offers valuable insights into the acceptance and attitudes towards AI integration in ophthalmology, it should be noted that the available data is primarily from the United States. To facilitate the effective and equitable implementation of AI technologies in ophthalmology, it would be of significant benefit to gain a more comprehensive understanding of the perspectives of diverse global stakeholders, including physicians, patients, and policymakers. To this end, it would be advantageous to expand the research to other regions, such as Europe and Switzerland, by conducting in-depth surveys of key stakeholders.

For successful AI adoption in ophthalmology, developers must align tools with clinical needs, focusing on user-friendly interfaces that cater to healthcare professionals of varying tech expertise. Effective integration requires collaboration with clinicians, iterative feedback loops, and regular real-world validation to ensure the AI meets practical demands and adapts to diverse patient populations. Developers should prioritize transparency in AI decision-making processes, aiming for understandable, explainable outputs that can be trusted by users. Additionally, compliance with ethical guidelines, patient data privacy, and equitable performance across demographics are vital for building credibility. Patient-centered designs remain a core aspect, ensuring that AI achieves technical accuracy, enhances patient outcomes, reduces diagnostic time, and improves overall care experiences. By addressing these factors, developers can create AI solutions that foster smoother integration, build trust among users, and ultimately advance patient care.

## Conclusion

This comprehensive analysis of the current landscape and stakeholder perspectives on integrating AI in ophthalmology illustrates the accelerated evolution of AI technologies for disease detection, treatment planning, and patient management in augmenting their expertise. However, stakeholders, including healthcare professionals and patients, have expressed concerns about the impact on the patient–clinician relationship, the need for continued education and training, the integration of XAI, securing interoperability with existing systems, and issues related to data privacy, costs, and global disparities in AI development and implementation. To realize the full potential of AI in ophthalmology, a collaborative approach involving multiple stakeholders must be adopted to address these concerns and foster the seamless integration of transformative technologies that can improve eye care delivery worldwide.
